# Changes in fibrinogen availability and utilization in an animal model of traumatic coagulopathy

**DOI:** 10.1186/1757-7241-21-56

**Published:** 2013-07-17

**Authors:** Jostein S Hagemo, Jørgen J Jørgensen, Sisse R Ostrowski, Anders Holtan, Yngvar Gundersen, Pär I Johansson, Pål A Næss, Christine Gaarder

**Affiliations:** 1 Department of Research, Norwegian Air Ambulance, Drøbak, Norway; 2 Department of Anaesthesiology, Oslo University Hospital, Oslo, Norway; 3 Department of Vascular Surgery, Oslo University Hospital, Oslo, Norway; 4 Department of Traumatology, Oslo University Hospital, Oslo, Norway; 5 Section for Transfusion Medicine, Capital Region Blood Bank, Rigshospitalet, University of Copenhagen, Copenhagen, Denmark; 6 Norwegian Defence Research Establishment, Kjeller, Norway; 7 Department of Surgery, University of Texas Medical School, Houston, TX, USA

**Keywords:** *Trauma*, *Shock*, *Coagulopathy*, *Fibrinogen*, *Thrombelastometry*

## Abstract

**Background:**

Impaired haemostasis following shock and tissue trauma is frequently detected in the trauma setting. These changes occur early, and are associated with increased mortality. The mechanism behind trauma-induced coagulopathy (TIC) is not clear. Several studies highlight the crucial role of fibrinogen in posttraumatic haemorrhage. This study explores the coagulation changes in a swine model of early TIC, with emphasis on fibrinogen levels and utilization of fibrinogen.

**Methods:**

A total of 18 landrace pigs were anaesthetized and divided into four groups. The Trauma-Shock group (TS) were inflicted bilateral blast femoral fractures with concomitant soft tissue injury by a high-energy rifle shot to both hind legs, followed by controlled exsanguination. The Shock group (S) was exposed to shock by exsanguination, whereas a third group was exposed to trauma only (T). A fourth group (C) served as control. Physiological data, haematological measurements, blood gas analyses and conventional coagulation assays were recorded at baseline and repeatedly over 60 minutes. Thrombelastometry were performed by means of the tissue factor activated ExTEM assay and the platelet inhibiting FibTEM assay. Data were statistically analysed by repeated measurements analyses method.

**Results:**

A significant reduction of fibrinogen concentration was observed in both the TS and S groups. INR increased significantly in the S group and differed significantly from the TS group. Maximum clot firmness (MCF) of the ExTEM assay was significantly reduced over time in both TS and S groups. In the FibTEM assay a significant shortening of the clotting time and an increase in MCF was observed in the TS group compared to the S group.

**Conclusion:**

Despite a reduction in clotting capability measured by ExTEM MCF and a reduced fibrinogen concentration, extensive tissue trauma may induce an increased fibrin based clotting activity that attenuates the hypocoagulable tendency in exsanguinated animals.

## Background

Disturbances of haemostasis are frequently encountered in the trauma setting. Measured by conventional coagulation tests, coagulopathy is found in 10-34% of patients on arrival in hospital [1]. The presence of trauma-induced coagulopathy (TIC) is associated with a four-fold increase in mortality [2]. Loss and consumption of coagulation factors, plasma dilution, hypothermia and acidaemia are known to be important causes of coagulopathy [3]. However, several studies identify the combination of hypoperfusion and tissue injury as primary drivers of TIC prior to the onset of hypothermia and acidaemia, and independent of iatrogenic dilution [2,4-6].

The exact pathophysiologic mechanisms contributing to TIC are insufficiently described. Some studies suggest that an abnormal expression of thrombomodulin on the endothelial surface activates excessive amounts of protein C, which in turn diverts haemostasis in the direction of hypocoagulability and hyperfibrinolysis [7,8]. Opponents of this theory argue that coagulopathy in trauma is merely a fibrinolytic phenotype of disseminated intravascular coagulopathy, as can be seen in any type of patients with serious hypoperfusion [9]. Others focus on endothelial cell and glycocalyx damage as critical components of TIC [10].

Regardless of the mechanism of TIC, available fibrinogen seems to play a pivotal role, as it is the end substrate for fibrin formation, and subsequent clot formation [11]. Several authors advocate early fibrinogen substitution in the massively bleeding trauma patient [12-14]. A number of retrospective studies support a 1:1 ratio of fresh frozen plasma (FFP) to packed red blood cell (PRBC) in massive transfusion [15-17], although this strategy may have serious adverse effects in the post resuscitation phase, especially in the hypercoagulable patients [18]. Fibrinogen concentrates and cryoprecipitate have emerged as alternative sources, but their role in massive transfusion still lacks firm evidence.

In the absence of conclusive clinical trials, a number of animal experiments have been conducted to elucidate the mechanisms of traumatic coagulopathy. However, only a few of these include massive tissue injury, and a minority describe changes in haemostasis occurring prior to iatrogenic dilution. In this study we aim to develop a large animal model to characterize the immediate changes in coagulation, focusing more specifically on fibrinogen availability and utilization following the combination of massive tissue injury and severe hypoperfusion.

## Methods

### Animals, anaesthesia and monitoring

The study was approved by the Norwegian Animal Research Authority. All animals received care in strict compliance with the Animal Welfare Act and statutes from the Norwegian Ministry of Agriculture. The anaesthetic and analgesic regimen used in the study was in accordance with a current protocol for surgical training on animals.

Landrace pigs (40–69 kg) were sedated by an intramuscular injection of 0,06 mg/kg medetomidin hydrochloride (Clinipharm, Switzerland) and 3,0 mg/kg tiletamin/zolazepam (Boehringer Ingelheim, Germany). An intravenous dose of 0,2 mg/kg butorphanol tartrate (American Home Products Corporation, USA) was administered for analgesia. After induction, the animals were intubated and hand-ventilated. In order to secure adequate analgesia, a single shot of 0,15 ml/kg lidocaine 2% was administered epidurally in the lumbosacral region. Cannulation of the left carotid artery and left jugular vein was performed with a 16G cannula for monitoring, blood sampling and controlled exsanguination during the experiment. Anaesthesia was maintained with 2% isofluorane (Baxter International Inc, IL, USA), and the animals were normoventilated to a target pCO_2_ of 5.0-6.0 kPa with FiO_2_ 0,5. 500 ml of normal saline was administered intravenously over 60 minutes from induction of anaesthesia. All animals were monitored with intra-arterial blood pressure, ECG, SpO_2_, and a pharyngeal temperature probe. At the end of the experiment, the animals were euthanized by an intravenous dose of 4 g of Thiopental sodium (Hospira Inc. IL, USA).

### Procedure

Eighteen animals were randomly divided into four groups. One group was exposed to both trauma and haemorrhagic shock (TS group, n = 8). One group underwent shock without injury (S group, n =4). One group was inflicted massive tissue injury but not exposed to shock (T group, n = 4), and finally one group, that was only anaesthetized and instrumented, served as control (C group, n = 2).

Tissue injury was created by two standardized high-energy rifle shots to both hind legs, creating bilateral blast femoral fractures with significant concomitant soft tissue injury [19]. The wounds were packed immediately for haemorrhage control, and an estimate of immediate blood loss was recorded. Exsanguination was induced by controlled extraction of blood at 1 ml/kg/min, aiming at a total blood loss of 30 ml/kg over 30 minutes, equivalent to about 45% of the estimated total blood volume (67 ml/kg). Blood extraction was terminated if a mean arterial pressure (MAP) < 30 mmHg was reached. If the volume substitution in excess of the above mentioned 500 ml normal saline was required to avoid circulatory collapse, the animal was excluded from the study.

### Sampling and measurements

Blood pressure, heart rate, oxygen saturation and amount of blood extracted were recorded every 5 minutes. Blood samples were drawn at baseline (pre injury), 0 minutes (immediately after injury), and after 30 and 60 minutes. Samples for haemoglobin concentration (Hgb) and platelet count (Plt) were collected in ethylenediaminetetraacetic tubes and performed on an Advia 60 Hematology System (Bayer Healthcare LLC, Tarrytown, NY, USA). Samples for coagulation assays were collected in citrated tubes. Fibrinogen, INR and aPTT assays were performed on a STA-R Evolution analyzer (Diagnostica STAGO, Inc, Parsippany, NJ, USA). Samples for RoTEM (TEM International GmbH, Munich, Germany) analyses were recalcified and activated by tissue factor in the ExTEM assay. For the FibTEM assay platelets were inhibited by cytochalasin D, in order to isolate the fibrin based fraction of the blood clot. Blood for arterial blood gas analyses were collected in heparinized syringes and analysed on an OPTI Critical Care Analyzer AVL (Roche Diagnostics Cooperation, Indianapolis, IN, USA).

### Statistical methods

Statistical analysis was performed using SAS 9.1 (SAS Institute Inc., Cary, NC, US). Repeated-measures analyses were performed using a means model (PROC MIXED, SAS) assuming compound symmetry covariance structure among the repeated measurements. The model included group (S group and TS group), time (baseline, 0, 30, 60 min) and group*time effects. The T group and C groups were not included in this model since the primary focus of the study was to study TIC, of which shock is a critical component. Significant group or time effects were analyzed by two-sample or paired post hoc t-tests. One-way ANOVA was used for analyses of difference in body weight and total bleeding between groups. Two-sample t-tests were used to detect changes between groups at baseline. Data are presented as means (SD). P-values <0.05 were considered significant.

## Results

### Measurements of blood loss and shock

Of the 18 animals included in the experimental groups, 16 completed the protocol. Two animals in the TS group were excluded due to circulatory collapse. There were no significant differences in body weight between groups (p = 0.590). Temperature remained above 37.2 in all subjects, and did not change significantly from baseline to 60 minutes in any of the groups (p = 0.134 for all groups combined). In the TS group the mean total amount of blood loss was 25.7% (SD: 7.9%), and did not differ significantly from the S group with mean blood loss 34.7% (SD: 6.9%) (p = 0.34). In the T group a mean estimated blood loss of 4.1% (SD: 2.7%) as a direct result of the injury was observed. Table 1 summarizes physiological parameters measured in the four different groups. Mean arterial pressure was significantly reduced after 60 minutes in all groups compared to baseline, and was significantly lower in the TS group compared to the other groups. Base excess was significantly reduced compared to baseline only in the TS group, and differed significantly from the rest of the groups.

**Table 1:** Physiological parameters in the four different groups

		**Base line**	**0 minutes**	**30 minutes**	**60 minutes**
**MAP**	TS	125.3 (23.9)	71.6 (18.2)*	36.2 (3.0)*†	36.8 (5.6)*†
	T	122.3 (10.3)	66.7 (8.14)*	70.3 (14.1)*	68.3 (13.5)*
	S	94.8 (27.0)		44.8 (4.0)*†	53.3 (8.2)*†
	C	120.0 (7.8)		85.5(7.8)*	85.0 (7.0)*
**HR**	TS	85.3 (4.7)†	95.3 (12.1)	96.4 (12.0)	99.2 (17.6)
	T	91.3 (7.0)	92.0 (10.2)	88.5 (12.1)	87.6 (13.6)
	S	106.0 (12.1)†		91.8 (9.6)	92.3 (11.7)
	C	101.0 (5.0)		116.0 (7.1)	106.5 (2.1)
**BE**	TS	7.88 (2.49)	6.88 (2.44)	4.18 (4.24)	1.27 (3.63)*†
	T	9.38 (5.17)	7.85 (0.72)	6.10 (2.82)	6.65 (0.65)
	S	7.70 (1.43)		6.35 (1.13)	6.65 (0.65)†
	C	9.30 (0.42)		10.25 (1.63)	9.00 (0.99)

*TS* Trauma Shock group, *T* Trauma group, *S* Shock group, *C* Control group, *MAP* Mean arterial pressure, *HR* Heart rate, *BE* Base excess.

* indicates significant change from baseline measured by paired t-test.

† indicates significant difference between TS and S groups by two-sample t-test.

### Haematology and conventional coagulation parameters

**Figure 1:**
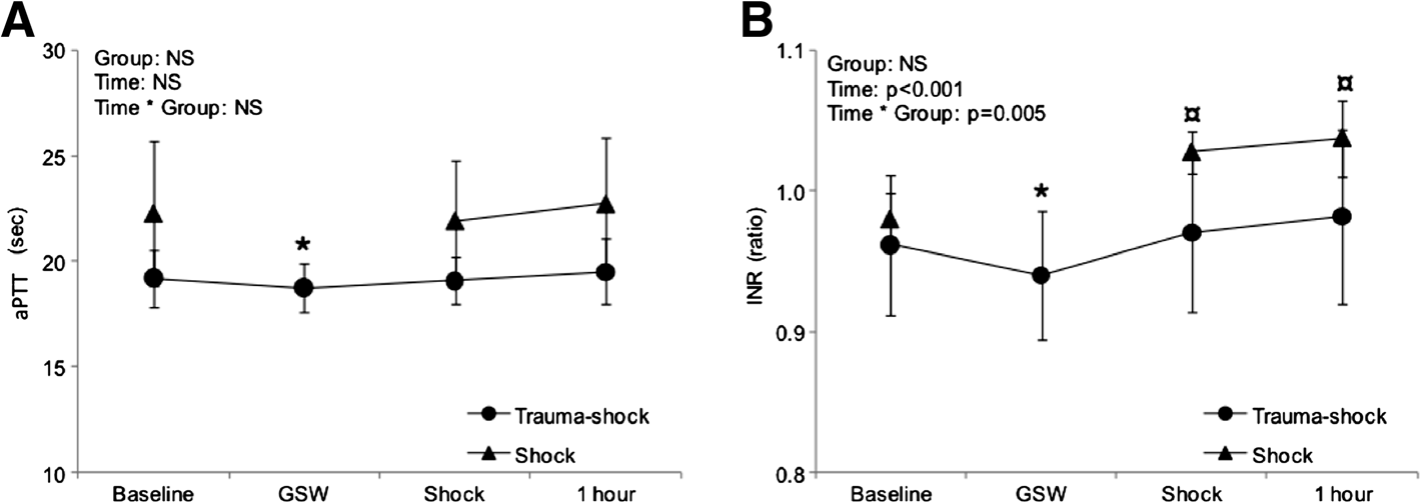
**Changes in activated partial thromboplastin time (aPTT) (A) and international normalized ratio (INR) (B) over time for Trauma-Shock (TS) and Shock (S) groups, with mixed-model statistical analyses.** * indicates significant difference from baseline in TS group. ¤ indicates significant difference from baseline in S group. GSW: gunshot wound.

**Figure 2:**
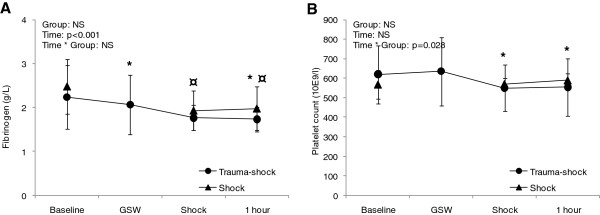
**Changes in fibrinogen concentration (A) and Platelet count (B) over time for Trauma-Shock (TS) and Shock (S) groups, with mixed-model statistical analyses.** * indicates significant difference from baseline in TS group. ¤ indicates significant difference from baseline in S group. GSW: gunshot wound.

Haemoglobin concentration and conventional coagulation parameters are summarized in Table 2. In the TS and S groups haemoglobin and fibrinogen concentrations fell significantly throughout the observation period, but did not differ significantly between the groups at any time. INR was significantly increased compared to baseline in the S group after 30 and 60 minutes, and also differed significantly from the TS group over time by mixed model analyses (Figure 1). In contrast, a reduction of both INR and aPTT compared to baseline was found in the TS group at time 0, shortly after injury (Figure 1). Platelet count was reduced after 30 minutes in the TS group and differed significantly from the S group by mixed model analyses (Figure 2).

**Table 2:** Haematological values and conventional coagulation tests

		**Base line**	**0 minutes**	**30 minutes**	**60 minutes**
**Hgb**	TS	10.67 (0.57)	9.95 (0.96)*	9.15 (1.32)*	8.98 (1.26)*
	T	10.65 (1.08)	9.75 (0.72)	9.58 (0.81)*	9.53 (0.46)
	S	10.35 (0.66)		8.83 (0.26)*	8.65 (0.41)*
	C	11.0 (0.35)		11.05 (0.35)	10.90 (0.14)
**Plt**	TS	619.5 (149.7)	636.0 (175.1)	549.5 (119.1)*	554.7 (146.3)*
	T	431.8 (110.9)	482.5 (144.5)	436.1 (76.6)	470.0 (98.6)
	S	568.0 (74.7)		570.5 (29.1)	589.3 (36.2)
	C	539.0 (241.8)		550.0 2410.4)	624.5 (334.5)
**Fib**	TS	2.24 (0.73)	2.07 (0.67)*	1.76 (0.29)	1.73 (0.29)*
	T	1.7 (0.07)	1.66 (0.10)	1.57 (0.11)	1.54 (0.11)
	S	2.48 (0.62)		1.93 (0.45)*	1.98 (0.5)*
	C	2.01 (0.04)		2.13 (0.06)	1.92 (0.07)
**INR**	TS	0.96 (0.05)	0.94 (0.05)*	0.97 (0.06)	0.98 (0.06)
	T	0.99 (0.03)	0.97 (0.03)	0.98 (0.03)	0.98 (0.02)
	S	0.98 (0.02		1.03 (0.01)*	1.04 (0.03)*
	C	0.98 (0.01)		0.98 (0.01)	1.00 (0.02)
**aPTT**	TS	19.2 (1.35)	18.7 (1.14)*	19.1 (1.11)	19.5 (1.55)
	T	19.5 (1.38)	19.0 (1.37)	19.1 (1.72)	19.3 (1.05)
	S	22.3 (0.92)		21.9 (2.84)	22.7 (3.17)
	C	21.5 (0.92)		21.3 (0.28)	22.2 (0.64)

*TS* Trauma Shock group, *T* Trauma group, *S* Shock group, *C* Control group, *Hgb* Haemoglobin, *Plt* platelet count, *Fib* Fibrinogen concentration, *INR* International normalized ratio, *aPTT* activated partial thromboplastin time.

* indicates significant change from baseline measured by paired t-test.

### RoTEM analyses

**Figure 3:**
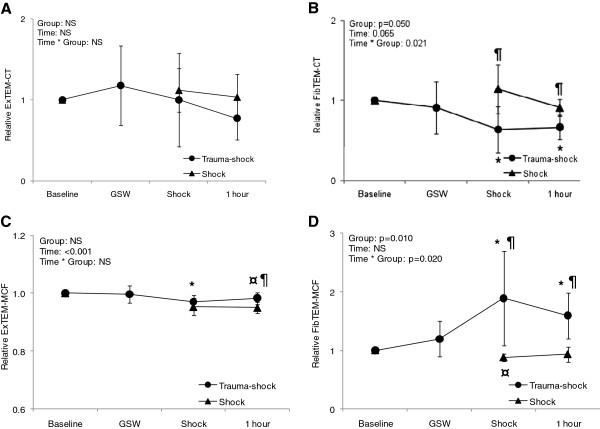
**Changes in RoTEM parameters over time as ratios of baseline, with mixed-models analyses. A**) ExTEM clotting time (CT). **B**) FibTEM CT. **C**) ExTEM maximum clot firmness and **D**) FibTEM MCF. * indicates significant difference from baseline in TS group. ¤ indicates significant difference from baseline in S group. ¶ indicates differences between groups by two sample t-test. GSW: gunshot wound.

Mean values and standard deviation of RoTEM analyses are given in Table 3. Due to a chance inequity of baseline values in the different groups, values for each subject was converted to relative values from baseline for further statistical analyses (Table 4). The ExTEM assay showed no significant changes in clotting time (CT). The maximum clot firmness (MCF) was significantly reduced in both the TS and S groups compared to baseline, but at different time points (30 and 60 minutes, respectively) (Figure 3). After 60 minutes the MCF in the S group was significantly lower compared to the TS group. By mixed model analyses, changes were significant over time, but not between the two groups.

**Table 3:** Coagulation analyses by RoTEM

			**Base line**	**0 minutes**	**30 minutes**	**60 minutes**
ExTEM	**CT**	TS	112.7(49.70)	113.7 (24.3)	91.2 (29.8)	77.7 (16.9)
		T	100.0 (23.8)	116.8 (39.2)	93.5 (22.5)	82.5 (15.6)
		S	66.5 (12.4)		72.3 (8.0)	66.3 (3.9)
		C	101.0 (41.0)		71.5 (10.6)	85.5 (5.0)
	**MCF**	TS	77.5 (3.9)	77.17 (3.2)	75.2 (3.2)	76.2 (4.3)
		T	75.0 (2.6)	74.8 (2.4)	71.0 (8.7)	73.0 (1.4)
		S	80.0 (2.5)		76.3 (3.8)	76.0 (2.2)
		C	75.5 (3.5)		77.0 (2.8)	76.5 (3.5)
FibTEM	**CT**	TS	120.3 (31.5)	103.0 (28.4)	69.5 (11.8)	76.8 (16.1)
		T	112.3 (56.4)	83.3 (24.6)	95.0 (19.1)	97.8 (47.5)
		S	69.6 (14.3)		77.0 (14.9)	62.5 (9.7)
		C	101.0 (41.0)		71.5 (10.6)	85.5 (5.0)
	**MCF**	TS	26.2 (23.6)	30.5 (24.9)	39.8 (18.0)	35.8 (19.7)
		T	35.3 (24.7)	40.3 (15.6)	25.8 (18.8)	35.0 (18.1)
		S	61.5 (6.5)		54.0 (6.8)	56.8 (4.0)
		C	49.0 (2.8)		49.5 (10.6)	48.0 (7.1)

*TS* Trauma Shock group, *T* Trauma group, *S* Shock group, *C* control group, *CT* Clotting time, *MCF* maximum clot firmness, Values are given as mean (SD).

**Table 4:** Coagulation analyses by RoTEM converted to ratios of baseline values

			**Base line**	**0 minutes**	**30 minutes**	**60 minutes**
ExTEM	**CT**	TS	1.0 (0.0)	1.18 (0.49)	1.0 (0.58)	0.77 (0.27)
		T	1.0 (0.0)	1.16 (0.29)	1.02 (0.49)	0.86 (0.26)
		S	1.0 (0.0)		1.12 (0.27)	1.04 (0.28)
		C	1.0 (0.0)		0.79 (0.43)	0.91 (0.32)
	**MCF**	TS	1.0 (0.0)	1.0 (0.03)	0.97 (0.02)*	0.98 (0.02) †
		T	1.0 (0.0)	1.0 (0.01)	0.95 (0.10)	0.97 (0.02)
		S	1.0 (0.0)		0.95 (0.03)	0.95 (0.02)*†
		C	1.0 (0.0)		1.02 (0.01)	1.01 (0.01)
FibTEM	**CT**	TS	1.0 (0.0)	0.91 (0.32)	0.64 (0.29)*†	0.66 (0.15)*†
		T	1.0 (0.0)	0.95 (0.63)	1.08 (0.67)	0.97 (0.55)
		S	1.0 (0.0)		1.14 (0.30)†	0.91 (0.11)†
		C	1.0 (0.0)		0.79 (0.43)	0.91 (0.32)
	**MCF**	TS	1.0 (0.0)	1.19 (0.30)	1.89 (0.81)*†	1.59 (0.39)*†
		T	1.0 (0.0)	1.45 (0.61)	0.93 (0.49)	1.15 (0.32)
		S	1.0 (0.0)		0.88 (0.07)*†	0.93 (0.13)†
		C	1.0 (0.0)		1.01 (0.16)	0.98 (0.09)

*TS* Trauma Shock group, *T* Trauma group, *S* Shock group, *C* Control group, *CT* Clotting time, *MCF* maximum clot firmness, Values are given as mean ratios of baseline values (SD).

* indicates significant change from baseline measured by paired t-test.

† indicates significant difference between TS and S groups by two-sample t-test.

With the added platelet inhibitor in the FibTEM assay, CT was significantly reduced in the TS group compared to baseline after 30 and 60 minutes. These changes differed significantly from the S group. The shortening of the clotting time was paralleled by a significant increase in MCF in the TS group, compared both to baseline values and the S group (Figure 3).

## Discussion

We report a significant reduction in ExTEM MCF in animals exposed to haemorrhage during the trial period (TS and S groups). Fibrinogen levels were also reduced in these two groups. Despite this reduction, the animals exposed to injury prior to exsanguination (TS group), had a markedly increased fibrin based clotting activity measured by a shortening of the clotting time and increased maximum clot strength in the FibTEM assay. Interestingly, INR and aPTT was also transiently affected in a hypercoagulable direction in these animals. A similar trend towards increased clotting activity measured by the FibTEM assay was observed in the group exposed to injury without subsequent exsanguination (T group). In contrast, an increased INR was noted in animals exposed only to haemorrhage.

Massive haemorrhage has previously been studied in swine models [20-24], but only a few have studied the effect of hypoperfusion in combination with soft tissue injury and fractures in the initial phase prior to fluid resuscitation [25,26]. White et al. studied the effect on conventional coagulation parameters as well as thrombelastography using TEG, in a model of injured and haemorrhaged swine. As in our study they found a marked reduction in fibrinogen levels, compared to the control group, and a reduction in the maximum amplitude of the clot strength. In one of the trials a significantly increased INR was noted after trauma and shock. Neither of these studies included assays specifically evaluating the clotting activity after inhibition of platelets similar to the FibTEM assay in our study.

Our study differs from most previous experimental studies on coagulopathy in that the extent of tissue injury inflicted is more extensive. In both studies by White et al., tissue trauma was induced by using a captive bolt pistol to cause a unilateral femoral fracture and soft tissue trauma. In our study, both thighs were inflicted injury from a high-energy rifle projectile, shattering the femur and causing significant soft tissue damage. Assuming the increased fibrin clotting capability is related to the extent of trauma, this may explain why an increase in INR in the TS group was not found in our study.

Our study demonstrates a significant group*time difference in INR values, between the TS group and the S group. As reported in several studies, INR measurements are sensitive to fibrinogen activity [21,27]. The group*time differences in INR may be a manifestation of the same increased clotting activity seen in the FibTEM assay for the TS group, caused by the extensive tissue trauma.

Increased fibrin clot formation has previously been described in a model of sheep undergoing intramedullary nailing. The mechanism behind this is not entirely understood, but an association with intravasation of intra-medullary fat has been demonstrated [28]. In a more recent paper by Huseby et al., coagulation markers were studied during intra-medullary reaming procedures in swine [29]. They found a transient elevation of the thrombin-antithrombin (TAT) complex as a surrogate marker for increased thrombin activity. The rise in TAT was unopposed by the fibrinolytic enzyme tissue plasmin activator (t-PA), thus resulting in an enhanced fibrin formation.

Several observational studies in human trauma patients describe a significant positive correlation between fibrinogen concentration and FibTEM MCF values, directly contrasting the findings in our study [30]. There may be several reasons for this discrepancy. Firstly, in human trials, fibrinogen levels in massively bleeding trauma patients are typically lower than in our study [30]. The reduced fibrin availability may be the limiting factor of clot formation. Secondly, acidaemia is frequently observed in human trauma patients, and the offset from pH optimum may affect the utilization of fibrinogen negatively. The pH value in our animals remained in the normal range throughout the experiment despite exsanguination. Thirdly, our study did not include analyses of factor XIII. This factor is known to be an important prerequisite in the inter-linkage of fibrin strands, and is found to be reduced in human trauma patients [31].

This study has limitations related to the limited number of animals in each group. A type II error in the analyses cannot be ruled out. We used cytochalasin D to inhibit platelets in the FibTEM assay in this study. The reliability of this method was questioned in one previous study, due to possible incomplete platelet inhibition [32]. The platelet count in our study was however not significantly different between groups, and it is therefore unlikely that this has imposed a systematic bias in our analyses. The anaesthetic agents used may have had implications for the findings in our study compared to what is found in a human trauma population. Blocking of adrenergic response and vasodilatation may have affected coagulation parameters, both due to direct catecholamine effect and hypoperfusion. An alternative anaesthetic agent such as ketamine to maintain catecholaminergic stimulation could possibly have had implications for the results. However, experience with ketamine in this setting is sparse, and for ethical reasons, a well-established anaesthetic regimen securing adequate analgesia was chosen.

In conclusion, our animal model of shock and trauma produced a hypocoagulable state measured by the ExTEM assay. Despite this reduction in coagulation capability, and a reduction in fibrinogen availability, a significant increase in fibrin clotting activity was found to attenuate the hypocoagulable effect of hypoperfusion in the presence of tissue injury. Further studies are required to clarify the role of fibrin activity for research on traumatic coagulopathy.

## Competing interests

The research group has received support from TEM international and Roche Diagnostics in the form of reagents and leasing of devices at reduced prices.

## Authors’ contributions

JSH, JJJ, AH, PAN and CG have made substantial contributions to conception and design of the study. JSH, JJJ, AH, YG, PAN and CG have contributed in the acquisition of data. JSH, SRO, PIJ, PAN and CG have been involved in data analyses and drafting of the manuscript. All authors have been involved in revising the manuscript. All authors read and approved the final manuscript.
